# The Relationship between Cardiac Magnetic Resonance-Assessed Replacement and Interstitial Fibrosis and Ventricular Arrhythmias in Hypertrophic Cardiomyopathy

**DOI:** 10.3390/jpm12020294

**Published:** 2022-02-17

**Authors:** Aleksandra Karabinowska-Małocha, Ewa Dziewięcka, Paweł Banyś, Małgorzata Urbańczyk-Zawadzka, Maciej Krupiński, Małgorzata Mielnik, Jacek Łach, Aleksandra Budkiewicz, Piotr Podolec, Łukasz Żydzik, Sylwia Wiśniowska-Śmiałek, Katarzyna Holcman, Magdalena Kostkiewicz, Paweł Rubiś

**Affiliations:** 1 Department of Cardiac and Vascular Diseases, Jagiellonian University Medical College, John Paul II Hospital, Prądnicka Street 80, 31-202 Krakow, Poland; ewa@dziewiecka.pl (E.D.); j.lach@szpitaljp2.krakow.pl (J.Ł.); ppodolec@interia.pl (P.P.); swisniowskasmialek@gmail.com (S.W.-Ś.); katarzyna.holcman@gmail.com (K.H.); magdalena.kostkiewicz@uj.edu.pl (M.K.); pawelrub@poczta.onet.pl (P.R.); 2 Department of Radiology, John Paul II Hospital, Prądnicka Street 80, 31-202 Krakow, Poland; p.banys@szpitaljp2.krakow.pl (P.B.); m.urbanczyk@szpitaljp2.krakow.pl (M.U.-Z.); maciej.krupinski@gmail.com (M.K.); m.mielnik@szpitaljp2.krakow.pl (M.M.); 3 Students’ Scientific Group on Heart Failure, at the Department of Cardiac and Vascular Diseases, Jagiellonian University Medical College, John Paul II Hospital, Prądnicka Street 80, 31-202 Krakow, Poland; abudzix@gmail.com (A.B.); llukas2@op.pl (Ł.Ż.)

**Keywords:** hypertrophic cardiomyopathy, LGE, myocardial fibrosis, ECV

## Abstract

Non-sustained ventricular tachycardia (nsVT) creates the electrical basis for sudden cardiac death (SCD) in hypertrophic cardiomyopathy (HCM). We aimed to evaluate the relationship between interstitial fibrosis on cardiac magnetic resonance (CMR) and nsVT in HCM. A total of 50 HCM patients underwent CMR with a 3 T scanner to determine the presence of replacement fibrosis expressed by late gadolinium enhancement (LGE), and interstitial fibrosis expressed by native T₁, post-contrast T₁, and extracellular volume (ECV). The incidence of nsVT was assessed by Holter monitoring. We detected nsVT in 14 (28%) out of 50 HCM patients. Replacement fibrosis expressed by LGE was present in 37 (74%) patients and only showed a trend towards a differentiation between the groups with and without nsVT (*p* = 0.07). However, the extent of LGE was clearly higher in the nsVT group (3.8 ± 4.9% vs. 7.94 ± 4.5%, *p* = 0.002) and was an independent predictor of nsVT in a multivariable regression analysis (OR 1.2; 95%CI 1.02–1.4; *p* = 0.02). No relationship was observed between interstitial fibrosis and nsVT. To conclude, it was found that it is not the mere presence but the actual extent of LGE that determines the occurrence of nsVT in HCM patients; the role of interstitial fibrosis remains unclear.

## 1. Introduction

Hypertrophic cardiomyopathy (HCM) is a common genetic myocardial disease with a prevalence of approximately 1:500, caused by mutations in sarcomeric genes [1,2,3]. Macroscopically, HCM is characterized by a non-dilated left ventricle (LV), various degrees of LV hypertrophy (LVH), and normal systolic function. At the cellular level, the typical features of HCM include areas of hypertrophied and disorganized (disarray) cardiac myocytes and widespread areas of fibrosis [4]. Most patients with HCM are minimally symptomatic [4,5]. However, approximately one-fifth of patients develop moderate-to-severe symptoms of chest pain, early fatigue, palpitations, syncope, etc., that result in five major pathologies: diastolic dysfunction (DD), LV outflow tract obstruction (LVOTO), an imbalance between the myocardial oxygen supply and demand, end-stage heart failure (HF), and arrhythmias, including atrial fibrillation and non-sustained ventricular tachycardia (nsVT) that may lead to sudden cardiac death (SCD) [6,7,8].

Cardiac fibrosis is common in HCM and is particularly responsible for DD, arrhythmias, and end-stage HF. Two types of cardiac fibrosis of differing pathologies and biological roles exist: namely local (replacement, scarring) and diffuse (interstitial, reactive) fibrosis [5]. Replacement fibrosis develops as a consequence of the death of local myocytes (necrosis, apoptosis), whereas interstitial fibrosis is caused by systemic processes, such as hypertension, inflammation, or genetic mutations. Both local and diffuse fibrosis coexist in HCM, as well as in many other cardiomyopathies.

Cardiac fibrosis can be diagnosed either invasively, by means of an endomyocardial biopsy, or by imaging methods. Among non-invasive methods, cardiac magnetic resonance (CMR) is considered the most optimal and is a validated tool for fibrosis assessment. After the administration of a gadolinium-based contrast agent, late gadolinium enhancement (LGE) imaging makes the identification of areas of local fibrosis possible. Currently, it is widely accepted that the quantification of the LGE area is the most preferred method as it allows for longitudinal measurements rather than a mere binary classification (e.g., LGE present or absent). Since the introduction of T1 parametric mapping, interstitial fibrosis can be evaluated [9]. Among all the T1-parametric indices, extracellular volume (ECV) is the one best suited to measure and quantify interstitial collagen expansion (fibrosis). In brief, ECV is measured by combining native and contrast-enhanced T1 maps of blood and myocardium and is typically expressed as a percentage (%).

To date, numerous studies have shown that the presence and size of LGE (i.e., replacement fibrosis) may be related to ventricular arrhythmia and SCD [10,11,12]. On the other hand, an association between ventricular arrhythmia (i.e., nsVT) with interstitial fibrosis (i.e., as assessed with T1 mapping) is far less studied. Given the fact that interstitial fibrosis is a common and potentially clinically relevant finding, its eventual role in arrhythmic risk stratification should be thoroughly investigated. Thus, the principal aim of the study was to evaluate the relationship between interstitial fibrosis, expressed as ECV, and nsVT in HCM patients.

## 2. Materials and Methods

### 2.1. Study Population

In this prospective, single-center, observational study, a total of 50 patients with a diagnosis of HCM was included. HCM was diagnosed on the basis of the current guidelines of the European Society of Cardiology (ESC) [13], defined as: ≥15 mm thickness of one or more of the LV wall segments which cannot be accounted for by the common causes of increased afterload (hypertension or aortic stenosis) or, in the case of first-degree relatives of HCM patients, as a thickness of LV ≥ 13 mm. We did not include patients with previously implanted cardiac devices, severely reduced kidney function (GFR < 30 mL/min), or infiltrative disease. Patients underwent diagnostic procedures, including laboratory tests, echocardiography, a six-minute walk test, electrocardiographic (ECG) Holter monitoring, and CMR. Echocardiographic examinations were performed on commercially available devices in accordance with the current European and American guidelines [14]. All of the patients gave their informed consent. The study was conducted in accordance with the Declaration of Helsinki, and prior to the study, the protocol was approved by the Jagiellonian University Ethical Committee.

### 2.2. Cardiac Magnetic Resonance

CMR exams were performed on a 3.0-T scanner (Magnetom Skyra, Siemens, Erlangen, Germany) at the time of inclusion. The analysis of the CMR studies was based on the guidelines of the Society of Cardiovascular Magnetic Resonance [15] and the Syngo. VIA software version VB 40 (Siemens, Erlangen, Germany) was used to conduct this analysis. Three long-axis (2-, 3-, and 4-chamber) slices and short-axis slices covering the LV were used to obtain steady-state free precession cine images. The CMR protocol consisted of cine CMR, native and post-contrast T1 mapping, and LGE imaging.

#### 2.2.1. Assessment of Replacement Fibrosis

Approximately 15 min after the intravenous administration of 0.1 mmol/kg of body weight of gadolinium-based contrast agent, the short-axis LGE images were acquired sequentially. The presence of LGE in both the short axis and adequate perpendicular images indicated the presence of fibrosis. A threshold of 5 standard deviations in subsequent short-axis slices was used to assess the quantitative extent of LGE, and its value was stated as a percentage of the total LV mass [15].

#### 2.2.2. Assessment of Interstitial Fibrosis

T1 mapping was performed using (Siemens Skyra VE11 with MyoMaps) a Modified Look Locker Inversion (MOLLI) Recovery sequence before, and 15 min after, a gadolinium-based contrast agent injection. The following parameters of this sequence were used: breath-hold TR/TE of 281/1.1 ms, slice thickness of 8 mm, matrix of 144 × 256 pixels, FOV from 320 × 260 mm^2^, and a flip angle of 35°. Drawing regions of interest (ROI) in the mid-wall regions of each myocardial segment according to the AHA 16-segment model determined the native and post-contrast T1 values. To measure T1 blood pools, drawings from the center of the LV cavity were used. *ROIs* were copied between the pre- and post-contrast T1 maps. Artifact segments were not included. The means of all segments native and post-contrast T1 times was the global value. The ECV was computed by the following formula [15]: ECV = ((1/(post-contrast T1) − 1/(native T1))/(1/(blood post-contrast T1)) − 1/(blood native T1))*(1 − Hct).

### 2.3. Electrocardiographic Examinations

During the index visit, patients also underwent 48 h ECG Holter monitoring (Spacelabs Healthcare, Reynolds Medical, Lifecard CF, Snoqualmie, DC, USA). The analysis was conducted by two experienced technicians and supervised by a cardiologist. Ventricular tachycardia (VT) was defined as three or more consecutive ventricular beats at a rate greater than 100 beats/min [16]. All recorded VTs were non-sustained, which was defined as having a duration of less than 30 s.

### 2.4. Statistical Analysis

Results are presented as percentages (counts) or mean ± standard deviations. The normal distribution of quantitative variables was assessed using the Shapiro–Wilk test. Qualitative variables were compared with the chi-squared test, and quantitative ones with the t-Student or U-Mann–Whitney test according to the analysis of the normal distribution. All parameters differentiating patients with and without nsVT with *p*-values < 0.05 (Table 1 and Table 2) were included in the regression analyses. For two closely related variables, one was used in the regression model. Uni- and multivariable logistic regression models analyzed the associations between the analyzed parameters and the presence of nsVT. When the *p*-value was <0.05, the results were considered statistically significant. The statistical analysis was conducted with the Statistica package, version 13.0 (StatSoft, TIBCO Software Inc., Palo Alto, CA, USA).

## 3. Results

### 3.1. Baseline Characteristics

Based on 48 h Holter data, patients were classified into those with (*n* = 14; 28%) and without nsVT (*n* = 36; 72%). Patients with nsVT had a significantly higher value of body mass index, experienced dyslipidemia more often, and had a higher estimated 5-year risk of SCD, a larger left atrium (LA) diameter and LA volume index, and a higher E/e’ ratio. We did not observe differences in the levels of NT-proBNP and high-sensitive troponin T. Moreover, the patients did not differ from each other in terms of the applied pharmacotherapy (Table 1).

### 3.2. nsVT and CMR Data

**Figure 1:**
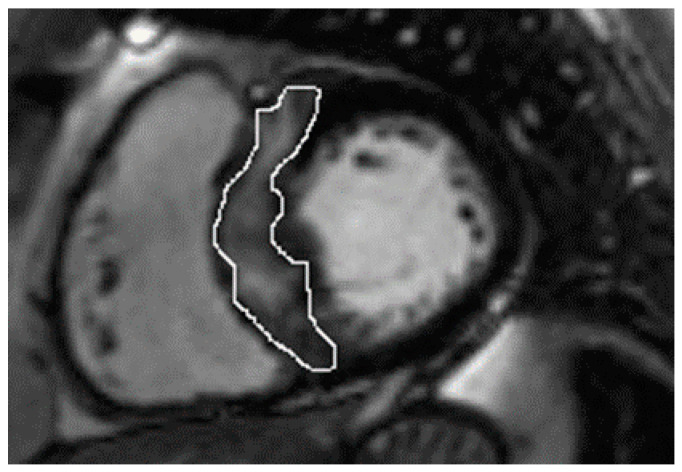
Late gadolinium enhancement (LGE) images show the quantification of the septal burden of replacement fibrosis (circled) via the technique which uses a 5-standard-deviations threshold on consecutive short-axis slices.

**Figure 2:**
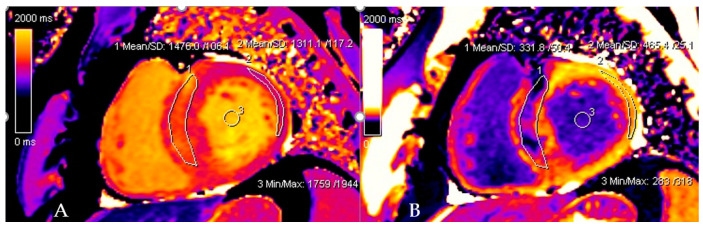
Native and post-contrast contours in the mid-myocardial area. The left ventricular cavity is shown (orange in native T1 mapping and dark blue in post-contrast T1 mapping) to enable the derivation of blood and myocardial T1 values. Standardized ROIs are placed in the septum to measure native (**A**) and post-contrast T1 times (**B**).

In the whole group, LGE was present in 37 (74%) patients. Patients with nsVT had a larger extent with respect to LGE (Table 2). However, the patients did not differ in native and post-contrast T1 times and ECV values, and the mere presence of LGE only showed a trend towards significance. Figure 1 and Figure 2 present examples of images obtained in the CMR studies.

### 3.3. Predictor Factors for nsVT

Among all the parameters differentiating patients with and without nsVT, univariable regression analysis presented a significant association between nsVT and LA diameter, and E/e’ and LGE extent (Table 3). However, in the multivariable regression model, only LA diameter and LGE extent were independently associated with nsVT (Table 3).

## 4. Discussion

The study findings can be summarized as follows: an independent association was found between replacement fibrosis (expressed as LGE extent and LA diameter) with nsVT. On the other hand, parameters that quantify interstitial fibrosis, such as native and post-contrast T1 times and ECV, were not found to be related to ventricular arrhythmias.

### 4.1. nsVT Predictors

#### 4.1.1. Replacement Fibrosis

Previous CMR studies have revealed the presence of LGE in approximately 70% of HCM patients [17,18], including those who are oligo- and asymptomatic [19]. In our group, LGE was present in 74% of patients, which is in line with previous observations. However, in some reports, the presence of LGE has ranged from 41% [20] to 90% [21]. Demonstrating the presence of LGE in the majority of patients with HCM, including the asymptomatic ones, sparked numerous studies investigating its relationship with prognoses and risk stratification. For the same reason, the quantitative analysis of LGE extent and its predictive value has also been investigated. While the evaluation of the presence of LGE is relatively simple, quantitative assessment is more complex, requires appropriate software, and is not standardized [22]. Although the bulk of studies have demonstrated a relationship between LGE and SCD risk, there are also reports that question these associations. Chan et al. in a study involving 1293 patients, demonstrated a significant relationship between LGE extent and the risk of SCD events [23], which was also confirmed in the meta-analysis by Weng et al., which included seven studies and 2993 patients [24]. Moreover, Weissler-Snir et al. showed a relationship between LGE extent and nsVT in HCM patients [25]. By way of contrast, Briasoulis et al., in his meta-analysis of six studies, showed that while there is a significant relationship between the mere presence of LGE and an increased risk of SCD in non-high-risk patients, the LGE extent was not significantly related to the risk of SCD [26]. Beyond this, Maron et al. showed no relationship at all between LGE and adverse cardiovascular events (SCD, appropriate implantable cardioverter-defibrillator discharge, and progressive HF symptoms) [27]; and Green et al., in his meta-analysis, only showed a trend towards significance in predicting SCD [28]. Although there are studies that have shown a significant relationship between the LGE extent and the arrhythmic endpoint in univariable analysis, this relationship was not confirmed in their multivariable analysis [29,30].

Importantly, we demonstrated an association between LGE extent and nsVT, evaluated at the same time (as CMR and Holter were performed almost simultaneously—within 1–2 days), which is dissimilar to most studies that have analyzed CMR and Holter at various time points, including several months apart or at unknown time intervals [31,32,33]. This may have some important implications. Given that the patients were in the same condition and being treated with stable therapies, it is probable that, as the clinical status changes (e.g., there is an exacerbation of the disease or medication is not taken), the arrhythmic risk is also probably changing. Despite the fact that this subject is poorly investigated, there is a likelihood that the amount of replacement fibrosis (i.e., LGE extent) is not a static pathology but varies over time. According to our observations, it was not merely the presence of LGE but its extent that was found to have diagnostic value for nsVT risk stratification. This is a finding that is consistent with a number of other studies [31,32].

Regarding the use of the LGE value as a factor in SCD risk stratification, there is a discrepancy between the American and European approaches [13,22]. The older European HCM risk score model takes into account the following parameters: age, maximum LV wall thickness, LA dimension, maximum LVOT gradient, the presence of nsVT, a family history of SCD, and unexplained syncope. Crucially, it fails to take into account the presence or extent of LGE. On the other hand, on the basis of several papers from Chan et al., Weng et, al., and Mentias et al. [23,24,34], the American College of Cardiology and American Heart Association Joint Committee issued a recommendation that extensive LGE be taken into consideration as a risk factor for potentially life-threatening ventricular arrhythmias [22]. Chan et al., as well as proving that the risk of SCD increased substantially with LGE levels at ≥15% of the LV mass, emphasized the linear relation between the %LGE and SCD event risk. Moreover, Chan et al. did not observe a significant increase in SCD in HCM patients with minimal LGE (1–5%) compared with those with no LGE [23]. In the work of Mentias et al., the study risk of primary events, consisting of SCD and appropriate implantable cardioverter-defibrillator (ICD) discharge, increased when %LGE was ≥15% [34]. In our study, we report lower values of LGE extent, as it was almost 8% in the nsVT-positive group in comparison to 3.8% in the nsVT-negative group; the difference is statistically sound and clearly indicates that more fibrotic LV is more prone to ventricular arrhythmias. A lack of consensus on the optimal quantitative method may be a source of variance in the results, as cited in the American guidelines [22].

#### 4.1.2. Left Atrium and nsVT

In many HCM patients, an enlarged left atrium is observed, which may be due to, among other causes, mitral regurgitation or DD [13]. Its size provides a lot of prognostic information [35,36] and is an independent risk factor for SCD included in the HCM risk score model [13,35]. In our observations, the enlarged size of the LA was an independent predictor of nsVT, a finding which is consistent with a previous study [25].

#### 4.1.3. Interstitial Fibrosis

Until recently, it was impossible to visualize and diagnose interstitial fibrosis, as conventional CMR only allows for the assessment of large areas of replacement fibrosis. Thanks to T1 parametric mapping, interstitial fibrosis can now be accurately assessed. As these two types of fibrosis have different pathologies, they most likely also have different clinical meanings, but this is an issue that is the subject of ongoing research.

As T1 mapping is a relatively new technology, only five studies analyzing the role of T1 times and ECV in the context of ventricular arrhythmia in HCM have been published so far. At present, the general picture emerging from these studies is unclear. Among these five studies, three reported some degree of association with ventricular arrhythmia. Levine et al. observed a significant two-fold increased prevalence of nsVT in patients with mean ECV above the study population mean of 27% compared with those with mean ECV < 27% [33]. The HCM group with nsVT or syncope in the study of Avanesov et al. had significantly higher global ECV than patients without nsVT or syncope, and the authors found that a cut-off value of ≥34% for global ECV resulted in a sensitivity of 88% and a specificity of 77% in the prediction of an increased SCD risk in HCM [32]. McLellan et al. showed that post-contrast ventricular T1 relaxation time was significantly reduced in patients with nsVT and patients with aborted SCD, and the quantity rather than the presence of LGE was associated with nsVT and aborted SCD [31].

Adding to the lack of clarity on the matter, the comparison of the ECV values between patients with and without nsVT by Chung et al. showed no statistically significant differences [37]. Furthermore, Mirelis et al. showed that ECV was not increased in ICD-implanted HCM patients with malignant ventricular arrhythmias vs. those without arrhythmias; however, in this study, the ECV was assessed by computed tomography [38]. In our group, the average ECV was approximately 28%, which is a figure that is slightly lower than that reported by Levine and Avanessov; nevertheless, septal ECV values were higher, reaching 30.5% in patients with documented nsVT. We observed no difference between native and post-contrast T1 times between patients with and without nsVT. As for ECV, which is a complex measure, involving myocardial and blood T1 times as well as hematocrit, there was no difference in terms of global ECV between patients with and without nsVT; still, it is worth noting that there was a numerical trend towards higher septal ECV values in nsVT patients compared to those without nsVT (30.5 ± 7.2 vs. 27.7 ± 5.6; *p* = 0.099). As fibrosis in HCM is most commonly present in the mid-septum, it may be that increased septal ECV is related to some level of arrhythmic risk.

The question of the relationship between ECV and nsVT remains unanswered, and our observations have not proved the value of ECV as a potential prognostic factor. In our opinion, the assessment of the relationship between ECV and ventricular arrhythmias and SCD requires multicenter studies with diverse populations and a standardized methodology.

An interpretation of the study results is offered here: LGE areas are large, disrupting myocyte organization, and exacerbating disarray. Large areas of scar tissue slow down the conduction-forming electro-micro circle loops. In contrast to replacement fibrosis, interstitial fibrosis is not induced by cell death and is a gradual process that can be reversed if the cause is treated promptly [39]. We can hypothesize that the gap junctions in diffuse fibrosis are not so far apart from each other, and therefore do not affect electrical impulse conduction to the same extent as is the case in irreversible replacement fibrosis.

### 4.2. Study Limitations

Our group was relatively small, especially after the division used. Therefore, the results should be interpreted with caution. Due to the small size of the subgroups, no analyses were performed, with the exception of drugs modifying the course of the fibrosis process. We only analyzed the effect of fibrosis on the Holter index and did not conduct a follow-up with extended duration. Larger multicenter studies with a long follow-up could provide valuable data.

## 5. Conclusions

In a contemporary cohort of HCM patients, an independent association was observed between a quantitative measure of replacement fibrosis—both LGE extent and nsVT. Conversely, T1 mapping derived measures of interstitial fibrosis; native and post-contrast T1 times and ECV were not related to nsVT. This study provides one further argument for the incorporation of LGE extent into a comprehensive SCD prognostic model. Moreover, further studies are needed to verify the role of interstitial fibrosis indices in SCD risk stratification in HCM, which currently remains undefined.

## Figures and Tables

**Table 1:** Baseline characteristics. Comparison of HCM patients with and without nsVT.

	Without nsVT (*n* = 36)	With nsVT (*n* = 14)	*p*-Value
Age (years)	48 (27.5)	54 (17)	0.3
Sex—male (*n*, %)	24 (66.7%)	11 (78.6%)	0.41
BMI (kg/m^2^)	29 ± 5.1	32.7 ± 6.6	0.042
LVOTO (*n*, %)	13 (36.1%)	6 (42.9%)	0.66
Diabetes mellitus (*n*, %)	3 (8.3%)	3 (21.4%)	0.2
Coronary artery disease (*n*, %)	5 (13.9%)	3 (21.4%)	0.51
Hypertension (*n*, %)	20 (55.6%)	9 (64.3%)	0.57
Atrial fibrillation (*n*, %)	3 (8.3%)	3 (21.4%)	0.2
Dyslipidaemia (*n*, %)	14 (38.9%)	10 (71.4%)	0.039
Syncope (*n*, %)	5 (13.9%)	2 (14.3%)	0.97
Family history of SCD (*n*, %)	4 (11.1%)	1 (7.1%)	0.67
Estimated 5-year risk of SCD (%)	2.09 (1.76)	5.8 (3.3)	<0.0001
NYHA class	1 (1)	2 (1)	0.28
SBP (mmHg)	127.8 ± 21.8	140.2 ± 18.17	0.07
6MWT-distance (m)	441 ± 121.1	411.2 ± 119.3	0.5
6MWT-Borg scale	3 (4)	2 (4.5)	0.9
LVEDd/BSA (mm/m^2^)	22.7 ±3.4	21.8 ± 3.6	0.43
MWT (mm)	19 (5.5)	21 (4)	0.098
LVEF (%)	65 (10)	68 (15)	0.34
LA (mm)	41 (7)	46 (11)	0.0007
LAVI (mL/m^2^)	39.5 (19.9)	59 (31.9)	0.01
E/e’	10 (5.6)	12.6 (8.6)	0.046
Max. LVOT gradient (mmHg)	21 (39.5)	37.5 (80)	0.47
RVSP (mmHg)	23.5 (11.5)	23 (12)	0.77
Hb (g/dL)	14.4 (1.9)	14.7 (1.9)	0.85
Hct (%)	41.6 ± 4.5	42.8 ± 3.8	0.3
hsTnT (ng/mL)	0.015 (0.017)	0.018 (0.014)	0.46
NT-proBNP (pg/mL)	444.5 (946.5)	671 (978)	0.33
BB (*n*, %)	31 (86.1%)	12 (85.7%)	0.97
Diltiazem/verapamil (*n*, %)	5 (13.9%)	2 (14.3%)	0.97
ASA (*n*, %)	7 (19.4%)	1 (7.1%)	0.29
ACEi/ARB (*n*, %)	15 (41.7%)	7 (50%)	0.59
MRA (*n*, %)	8 (22.2%)	6 (42.9%)	0.14
Loop diuretics (*n*, %)	10 (27.8%)	4 (28.6%)	0.96
Amiodarone (*n*, %)	1 (2.8%)	1 (7.14%)	0.48
OAC (*n*, %)	3 (8.3%)	3 (21.4%)	0.2
Statins (*n*, %)	15 (41.7%)	5 (35.7%)	0.67

Values are mean ± SD or median (IQR) or *n* (%). Abbreviations: BMI—body mass index, LVOTO—left ventricular outflow tract obstruction, SCD—sudden cardiac death, NYHA—New York Heart Association class, SBP—systolic blood pressure, 6MWT—6 min walk test, LVEDd—left ventricle end-diastolic diameter, BSA—body surface area, MWT—maximal left ventricle wall thickness, LVEF—left ventricle ejection fraction, LAVI—left atria volume indexed, E/E’ – ratio of early mitral inflow velocity to early mitral myocardial velocity, LVOT—left ventricular outflow tract, RVSP—right ventricular systolic pressure, Hb—hemoglobin, Hct—hematocrit, hsTnT—high-sensitive troponin T, NT-proBNP—N-terminal pro b-type natriuretic peptide, BB—beta-blocker, ASA—acetylsalicylic acid, ACEI—angiotensin-converting enzyme inhibitor, ARB—angiotensin receptor blockers, MRA—mineralocorticoid receptor antagonist, OAC—(VKA and non-VKA) oral anticoagulants.

**Table 2:** Comparison of CMR findings between HCM patients with and without nsVT.

	Without nsVT (*n* = 34)	With nsVT (*n* = 14)	*p*-Value
LVEF (%)	69 (11)	69.5 (10)	0.4
Svi (mL/m^2^)	51.5 ± 10.2	56.6 ± 9.7	0.1
LV mass (g)	190.9 ± 55	217.3 ± 46.2	0.13
LGE (*n*, %)	24 (66.7%)	13 (92.9%)	0.074
%LGE (%)	2.69 (5.47)	8.1 (7.36)	0.002
T1 native blood (ms)	1852.7 (111)	1842.7 (94.3)	0.5
T1 post-contrast blood (ms)	302 (44.7)	326.7 (57.3)	0.87
T1 native septal (ms)	1264 (91)	1305.6 (91.8)	0.43
T1 native global (ms)	1258.9 + 70.5	1275.1 ± 59.6	0.45
T1 post-contrast septal (ms)	489.5 (64)	487.5 (92)	0.52
T1 post-contrast global(ms)	471.2 ± 57	468.2 ± 53.4	0.87
ECV septal (%)	26.5 (5.2)	27.9 (5.4)	0.099
ECV global (%)	28.1 (6.2)	28.1 (4.8)	0.6

Values are mean ± SD or median (IQR) or *n* (%). Svi—stroke volume index, LGE—late gadolinium enhancement, %LGE—extent of LGE, ECV—extracellular volume, septal—mean value of 8- and 9-segment.

**Table 3:** Uni- and multivariable regression models for nsVT presence.

Parameter	Univariable OR (95% CI) *p*-Value		Multivariable OR (95% CI) *p*-Value	
Dyslipidemia	3.93 (0.99–15.5)	0.05	-	-
BMI	1.12 (0.997–1.269)	0.05	-	-
E/E’	1.21 (1.02–1.43)	0.024	1.19 (0.95–1.48)	0.1
LA	1.2 (1.06–1.36)	0.004	1.19 (1.03–1.38)	0.016
LGE extent	1.17 (1.02–1.35)	0.02	1.2 (1.02–1.4)	0.02

## Data Availability

The data presented in this study are available on request from the corresponding author. The data are not publicly available due to their clinical nature.
